# Bi-directional roles of IRF-1 on autophagy diminish its prognostic value as compared with Ki67 in liver transplantation for hepatocellular carcinoma

**DOI:** 10.18632/oncotarget.9365

**Published:** 2016-05-13

**Authors:** Hai-Ming Zhang, Shi-Peng Li, Yao Yu, Zhen Wang, Jin-Dan He, Yan-Jie Xu, Rong-Xin Zhang, Jian-Jun Zhang, Zhi-Jun Zhu, Zhong-Yang Shen

**Affiliations:** ^1^ First Central Clinical College, Tianjin Medical University, Tianjin, P. R. China; ^2^ Department of Transplantation, Tianjin First Central Hospital, Tianjin, P. R. China; ^3^ Tianjin Key Laboratory of Organ Transplantation, Tianjin, P. R. China; ^4^ Laboratory of Immunology and Inflammation, Tianjin Medical University, Tianjin, P. R. China; ^5^ Beijing Friendship Hospital, China Capital Medical University, Beijing, P. R. China

**Keywords:** IRF-1, Ki-67, autophagy, HCC, liver transplantation

## Abstract

The prognostic values of IRF-1 and Ki-67 for liver transplantation (LT) of hepatocellular carcinoma (HCC) were investigated, as well as the mechanisms of IRF-1 in tumor suppression. Adult orthotropic liver transplantation cases (*N* = 127) were involved in the analysis. A significant decreased recurrence free survival (RFS) was found in the Ki-67 positive groups. Ki-67, tumor microemboli, the Milan and UCSF criteria were found to be independent risk factors for RFS. In LT for HCC beyond the Milan criteria, a significant decrease in RFS was found in the IRF-1 negative groups. In SK-Hep1 cells, an increase in apoptosis and decrease in autophagy were observed after IFN-γ stimulation, which was accompanied with increasing IRF-1 levels. When IRF-1 siRNA or a caspase inhibitor were used, reductions in LC3-II were diminished or disappeared after IFN-γ stimulation, suggesting that IFN-γ inhibited autophagy via IRF-1 expression and caspase activation. However, after IRF-1 siRNA was introduced, a reduction in LC3-II was found. Thus basic expression of IRF-1 was also necessary for autophagy. IRF-1 may be used as a potential target for HCC treatment based on its capacity to affect apoptosis and autophagy. Ki-67 shows great promise for the prediction of HCC recurrence in LT and can be used as an aid in the selection of LT candidates.

## INTRODUCTION

Given the shortage of liver donors, effective use of liver donations represents an important medical and ethical issue. As one approach to address this problem, a number of requirements for an accurate evaluation regarding the outcome of liver transplantation (LT) for hepatocellular carcinoma (HCC) have been presented. However, due to the variable nature of liver tumors and differences in the origin and stage of these tumors, such evaluations remain a difficult and complicated process. LT for HCC has a distinct recurrence pattern from that of liver resection. Compared with intrahepatic metastasis, pulmonary metastasis is common. Even a recurrent lesion within a liver graft can also result from an extrahepatic metastasis. While a number of biomarkers, such as CK, CK19, GPC3, AFP, VEGF, EGFR, ERCC1, RRM1, TYMS, BRCA1, p53, VIM and Ki-67 are currently available for clinical assessment of tumor behavior, their prognostic value for LT of HCC is not clear. Moreover, upstream regulatory factors require further investigation for their effectiveness as markers and understanding of mechanisms of action.

Interferon regulatory factor 1 (IRF-1) is a transcriptional factor that mediates functions of interferon (IFN). IRF-1 has been referred to as the “master promoter”, due to its capacity to activate a variety of tumor suppressor genes. The anti-tumor effects of IRF-1 have been confirmed in a number of reports [1–4]. The current consensus on the mechanism of IRF-1 is that it activates a set of target genes involved in cell cycle regulation, apoptosis and immune responses. For example, IFN-γ induced cell cycle arrest and apoptosis are mediated by IRF-1 [5, 6].

Autophagy accounts for the majority of degradation of cellular constituents, which represents an important component for the maintenance of homeostasis. Some examples of essential proteins involved with autophagy include Beclin1, Atg5, Atg7 and LC3. While findings of a recent study have identified a pathway independent of Atg5 and Atg7, Beclin1 is still required for this Atg5/Atg7-independent autophagy [7]. Schmeisser H et al. reported that type I IFN was an inducer of autophagy in multiple cell lines. The capacity for IRF-1 to promote apoptosis and inhibit autophagy in splenocytes was demonstrated within endotoxemia [8], and a relationship between IRF-1 and autophagy was also reported in tumor cells. Schwartz-Roberts JL et al. identified ATG7 and Beclin1 as negative regulators of IRF-1 in human breast cancer cells [9]. Results from a recent study demonstrated that IFN-γ induced cell growth inhibition and cell death in HCC cells; and, IFN-γ induced autophagy instead of apoptosis in certain HCC cells through the IRF-1 signaling pathway [10]. Thus, the ability for IRF-1 to affect apoptosis and autophagy may represent one of its mechanisms involved with HCC suppression, but the exact roles of these processes require further investigation.

The results of our current ambispective cohort study show that Ki-67 was an independent risk factor for HCC recurrence after LT and the importance of IRF-1 in HCC recurrence has been demonstrated in patients with HCC beyond the Milan criteria. In a study with SK-Hep1 cells, apoptosis was enhanced and autophagy reduced after IFN-γ stimulation, an effect which was accompanied with an increase in IRF-1. IFN-γ is believed to inhibit autophagy via IRF-1 expression and Caspase activation as concluded from results obtained with siRNA and caspase inhibitor treatments. After introduction of IRF-1 siRNA, a reduction in LC3-II was also found. Thus, a basic expression of IRF-1 is also important for autophagy. Collating the findings of these reports suggests that IRF-1 may serve as a potential target for HCC treatment based on its modulatory effects upon apoptosis and autophagy; while Ki-67 may represent a valuable candidate for use in assessing LT.

## RESULTS

### Baseline data on clinical reports

**Figure 1:**
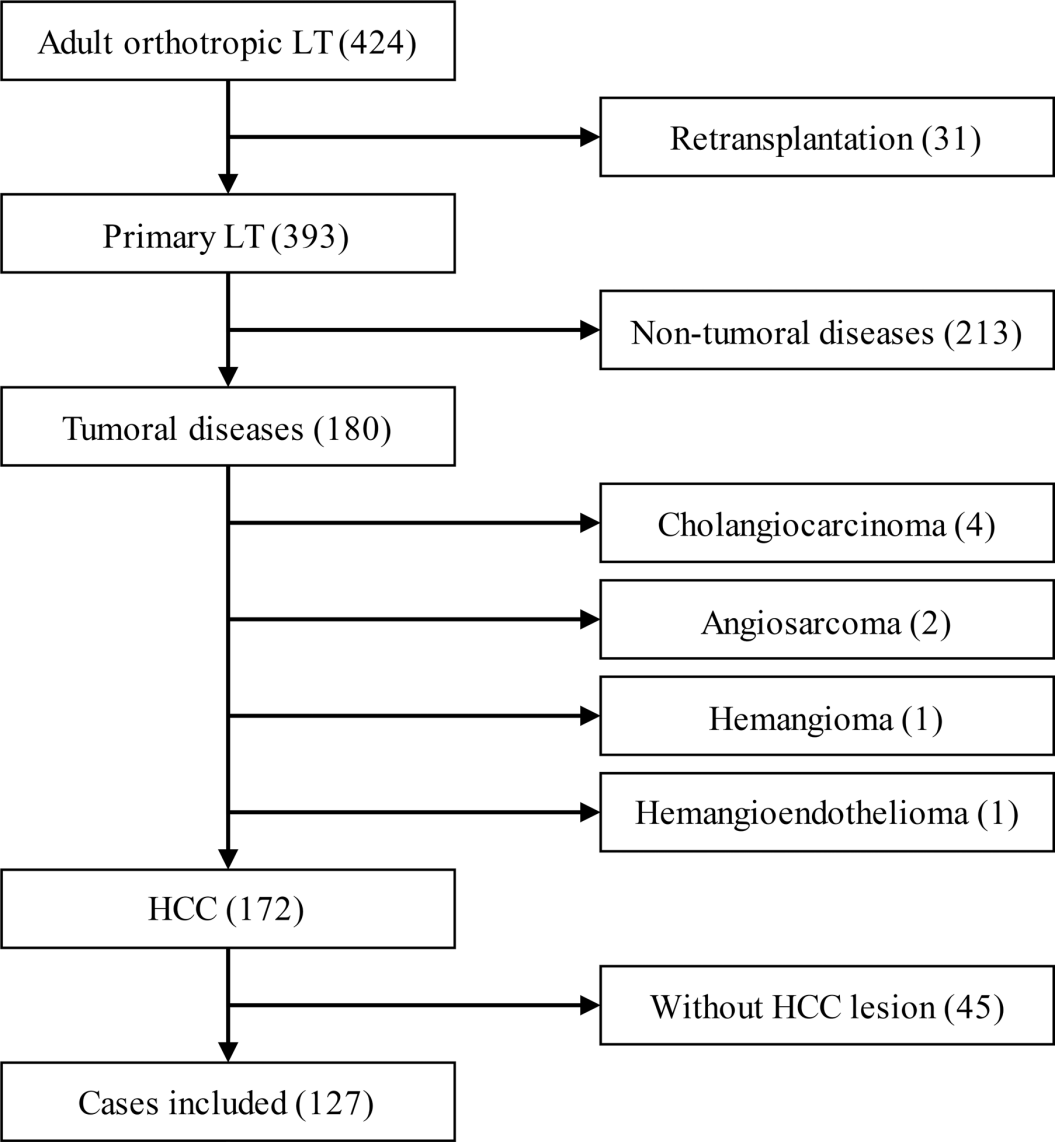
Numbers of cases included and excluded

From July 1st 2011 to July 1st 2014, 424 cases of adult orthotropic liver transplantation with whole liver grafts, including 31 cases of retransplantation, were performed at Tianjin First Central Hospital,. In the 393 cases of primary orthotropic liver transplantation, 213 cases with non-tumoral disease, 4 cases of cholangiocarcinoma, 2 cases of hepatic angiosarcoma, 1 case of hepatic hemangioma and 1 case of hepatic hemangioendothelioma were excluded from our study. The remaining 172 cases of LT for HCC complied with the 1, 2 and 3 items of inclusion criteria. Among the HCC patients, 45 cases (7 cases of T1, 34 cases of T2 and 4 cases of T3a, according to the imaging reports before downstaging therapy) had no suitable tumor tissue for analysis due to resection or other downstaging therapies before LT. As a result, 127 cases were involved in the final analysis (Figure 1).

Within these 127 cases of HCC, 102 patients were without any history of hepatectomy (primary HCC) while 25 had a history of hepatectomy (recurrent HCC). There were no significant differences between primary and recurrent HCC within clinicopathological factors as summarized in Table 1. All patients had liver cirrhosis, including 93 cases of post-hepatitis B liver cirrhosis and 24 cases of post-hepatitis C cirrhosis. HCC recurrence was present in 53 cases and there were 24 cases of death during the study, including 14 cases of death without HCC recurrence and 10 cases of death after HCC recurrence. For 7 of the death cases without HCC recurrence and 12 survival cases the follow-up period was less than 1 year. All 127 cases of HCC were involved in the analysis. The median follow-up time was 421 days (range = 9 to 1263 days).

**Table 1:** Clinicopathological characteristics of patients who underwent LT for HCC

	Primary HCC	Recurrent HCC	*P*
n	102	25	
Gender			0.285
Male	92	20	
Female	10	5	
Patient age, y (mean ± SD)	52.04 ± 7.97	52.24 ± 7.93	0.218
Couse of cirrhosis			0.135
HBV	73	20	
HCV	22	2	
Alcohol	2	0	
Cryptogenic	5	2	
PBC	0	1	
TNM stage			0.722
T1	14	5	
T2	39	7	
T3a	18	3	
T3b	19	6	
T4	12	4	
Milan and UCSF criteria			0.193
Within Milan criteria	34	8	
Between Milan and UCSF criteria	7	0	
Beyond UCSF criteria	61	17	
Differentiation			0.478
I~II	57	12	
III~IV	45	13	
Tumor microemboli			0.279
No	53	16	
Yes	49	9	
Median of follow-up (day)	427 (16~1263)	405 (9~886)	0.834

### Ki67 predicted recurrence free survival in LT for HCC

**Figure 2:**
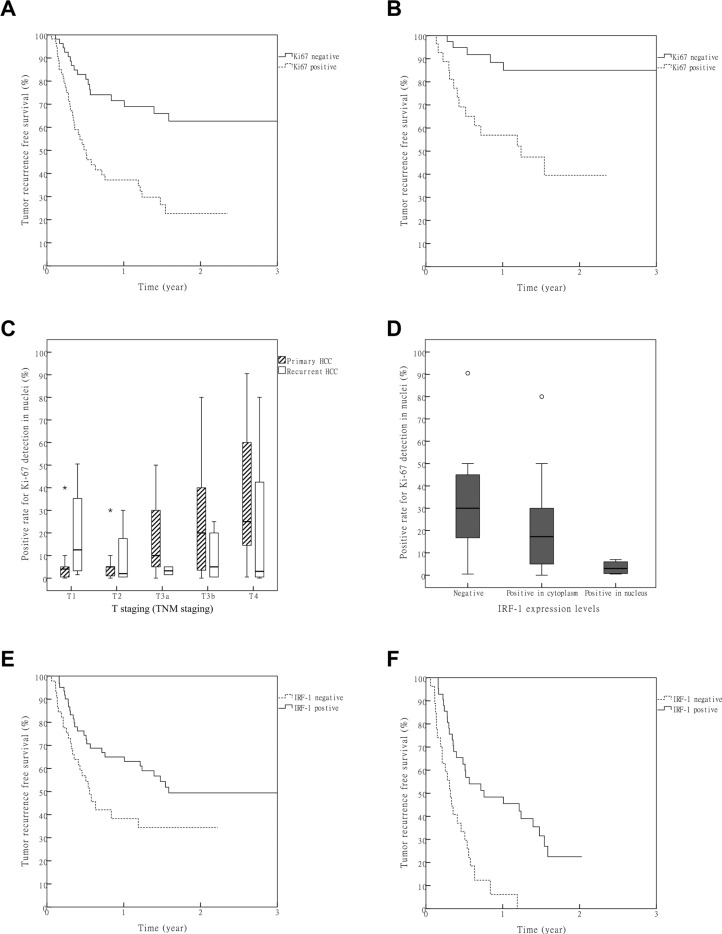
Value of Ki67 and IRF-1 in predicting post LT HCC recurrence (**A**) Statistically significant differences among all the patients were obtained for RFS between negative and positive groups of Ki67 (*P* = 1.6 × 10^−4^, Bonferroni correction α′ = 1.5 × 10^−3^). (**B**) Difference in RFS between negative and positive groups of Ki67 in the patients with T1-T3a HCC (*P* = 6.8 × 10^−4^). (**C**) A significant correlation was obtained between Ki-67 and T stage in the primary, but not recurrent, HCC group (Spearman correlation *R* = 0.459, *p* = 1.2 × 10^−5^ and *R* = −0.139, *P* = 0.527). *: Extreme outliers. (**D**) A significant negative correlation was obtained between IRF-1 and Ki-67 (Spearman correlation *R* = −0.405, *P* = 0.030). ○: Mild outliers. (**E**) Among all the patients, differences in RFSs between negative and positive groups of IRF-1 failed to achieve statistical significance after Bonferroni correction (*P* = 0.023, Bonferroni correction α′ = 1.5 × 10^−3^). (**F**) In patients with HCCs beyond the Milan criteria, a significant difference in RFS was found between the negative and positive groups of IRF-1 (*P* = 6.4 × 10^−5^, Bonferroni correction α′ = 1.5 × 10^−3^).

Cases were first stratified using the Milan-UCSF criteria and tumor microemboli. There was a significant difference in recurrence free survival (RFS) among patients with HCC within the Milan criteria, between the Milan and UCSF criteria and beyond the UCSF criteria (Table S2, *P* = 3.8 × 10^−14^). A significant difference also existed in RFS between patients with and without tumor microemboli (Table S2, *P* = 1.7 × 10^−11^). The immunochemical results of biomarkers are presented in Table S1. No significant differences were found between primary and recurrent HCC among these biomarkers. As expression levels of these biomarkers were highly variable, we grouped these biomarker expressions for two times: first, they were divided into negative and positive groups; then, they were redivided into low and high groups (Table 2). The RFS was compared between corresponding groups (negative vs. positive and low vs. high). Significant differences were found for Ki-67 within both the negative vs.positive and low vs.high groups (Figure 2A, Table 2, Table S2, *P* = 4.6 × 10^−5^and *P* = 1.6 × 10^−4^, respectively). In a subgroup analysis of patients with T1-T3a HCC, there was also a significant difference in RFS for the Ki-67 negative vs. positive group (Figure 2B, *P* = 6.8 × 10^−4^).

**Table 2:** Comparisons of RFSs between different expression groups of each molecule

Tumor marker	Grouping methods			
	Negative vs. positive group^a^		Low vs.high expression group^b^	
	Log Rank χ^2^	*P*	Log Rank χ^2^	*P*
CK	0.668	0.414	2.680	0.102
CK19	0.029	0.864	0.035	0.852
GPC3	0.364	0.547	1.423	0.233
AFP	0.417	0.518	1.436	0.231
VEGF	1.472	0.225	0.004	0.951
EGFR	0.587	0.444	0.257	0.612
ERCC1	0.448	0.503	1.955	0.162
RRM1	0.009	0.927	0.088	0.767
TYMS	0.019	0.891	2.622	0.105
BRCA1	0.678	0.410	4.322	0.038
p53	6.740	0.009	0.079	0.778
VIM	0.137	0.711	0.596	0.440
Ki-67	16.589	0.001^c^	14.268	0.001^c^
IRF-1	5.167	0.023	1.358	0.244

a Immunochemical results grouping: “−” was included in negative group; “+” or a higher expression was included in positive group.

b Immunochemical results grouping: “−” or “+” was included in low expression group; “++” or a higher expression was included in high expression group.

c Bonferroni correction, α′ = 1.5 × 10^−3^.

A Cox regression model was used to evaluate the independent predictive value of biomarkers. To reduce type II errors, all the biomarkers with *p* values less than 0.05 in Table 2 were analyzed in the model (backward LR, α_1_ = 0.05, α_2_ = 0.05). Included within these analyses were the TNM staging, Milan-UCSF criteria, tumor microemboli, BRCA1 (low/high group), p53 (negative/positive group), Ki-67 (positive rate of Ki-67 detection in nuclei) and IRF-1 (negative/positive group). Results of these analyses indicated that the Milan-UCSF criteria, tumor microemboli and Ki-67 were independent predictive factors for HCC recurrence after LT (Table 3, *P* = 1.37 × 10^−3^, *P* = 3.67 × 10^−4^ and *P* = 4.16 × 10^−4^). In subgroup analyses, a significant correlation between Ki-67 and T stage was found in the primary HCC group, but not in the recurrent HCC group (Figure 2C, Spearman correlation R = 0.459, *P* = 1.2 × 10^−5^ and R = −0.139, *P* = 0.527, respectively).

**Table 3:** Independent risk factors for HCC recurrence after LT

Factors	B	SE	Wald	df	Sig.	Exp(B) (95.0% CI)
Milan-UCSF criteria^a^	3.320	1.038	10.230	1	1.4 × 10^−3^	5.572 (1.906~16.288)
Tumor microemboli^b^	1.571	0.441	12.684	1	3.7 × 10^−4^	4.811 (2.027~11.420)
Ki-67^c^	2.565	0.727	12.447	1	4.2 × 10^−4^	13.007 (3.128~54.094)

a Tumors were grouped into 3 levels: within Milan criteria = 1, between Milan and UCSF criteria = 2, beyond UCSF criteria = 3;

b Tumors were divided into 2 groups: with and without tumor microemboli groups;

c Positive rate of Ki-67 detection in nuclei.

### IRF-1 predicted recurrence free survival in LT for HCC beyond the Milan criteria

There was a negative correlation between IRF-1 and Ki-67 (Figure 2D, Spearman correlation R = −0.405, *P* = 0.030). The difference in RFS between negative and positive IRF-1 expression groups did not achieve statistical significance among all the patients, after Bonferroni correction (Table 2, Figure 2E, *P* = 0.023, Bonferroni correction α′ = 1.5 × 10^−3^). To corroborate the findings indicating a correlation between IRF-1 expression and HCC recurrence, we compared the RFSs of different IRF-1 expression groups in patients with HCC beyond the Milan criteria. After exclusion of patients with HCC within the Milan criteria, a significant difference in RFSs was found between the IRF-1 negative and positive groups (Figure 2F, *P* = 6.4 × 10^−5^).

### IFN-γ promotion of apoptosis in SK-Hep1 cells was accompanied with increasing levels of IRF-1 and pSTAT1

**Figure 3:**
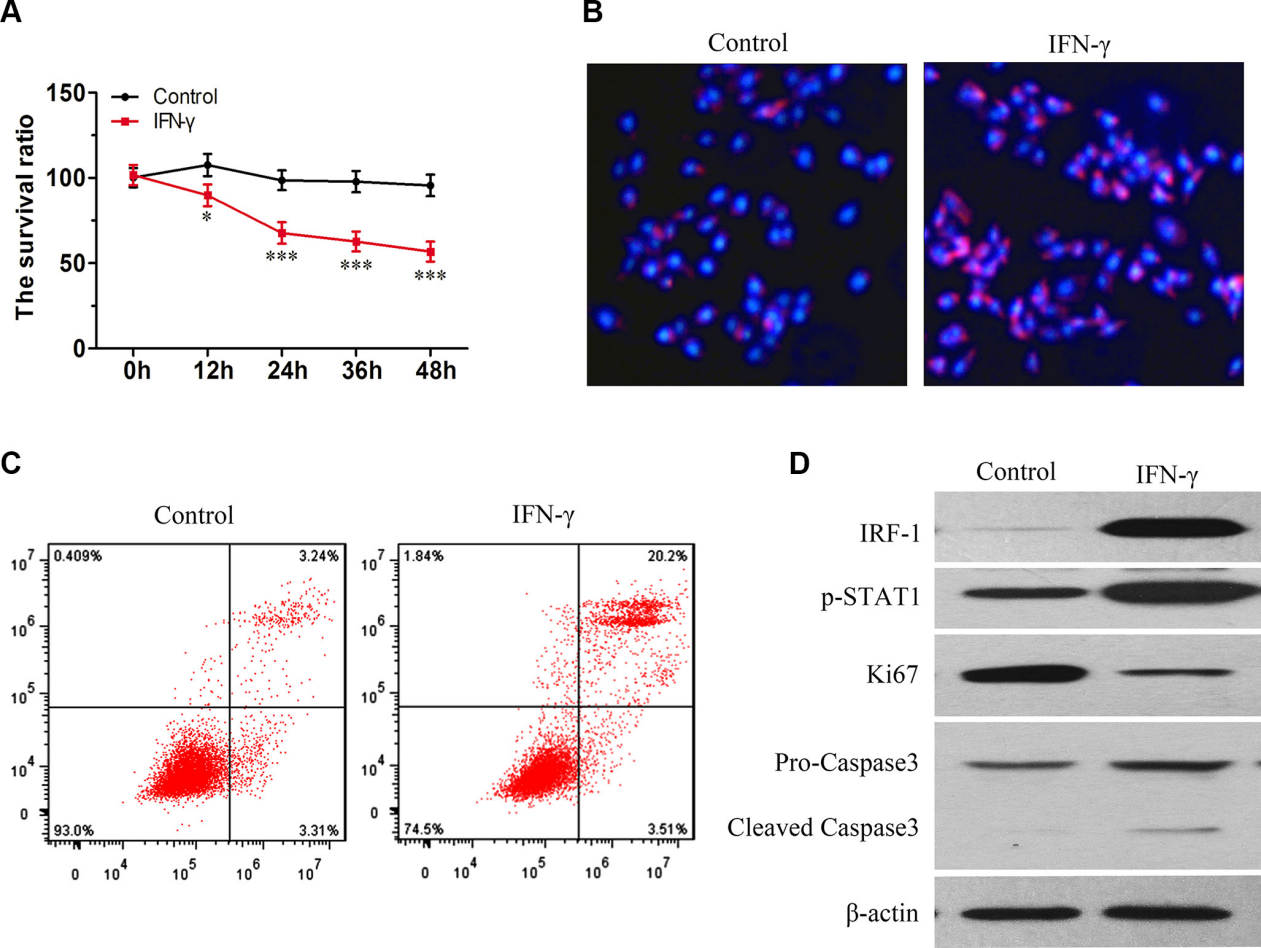
IFN-γ induced apoptosis in SK-Hep1 cells is associated with increasing levels of IRF-1 and pSTAT1 (**A**) Viability of living cells was decreased as a function of time in the IFN-γ group (mean ± SD; *P < 0.05; ***P < 0.001). (**B**) and (**C**) Levels of apoptosis increased after IFN-γ treatment. (**D**) As compared with the control group, levels of IRF-1, pSTAT1 and cleaved Caspase-3 increased while levels of Ki67 decreased.

In SK-Hep1 cells, the viability of living cells was decreased as a function of time following IFN-γ stimulation (Figure 3A). PI staining also showed an increased amount of apoptosis in the IFN-γ group (Figure 3B, Figure 3C). As compared with the control group, levels of IRF-1, pSTAT1 and cleaved Caspase-3 were increased in the SK-Hep1 cells stimulated with IFN-γ, as determined using Western Blot tests (Figure 3D). Levels of Ki-67 were decreased in the IFN-γ group (Figure 3D).

### IFN-γ suppressed autophagy via IRF-1 in SK-Hep1 cells

**Figure 4:**
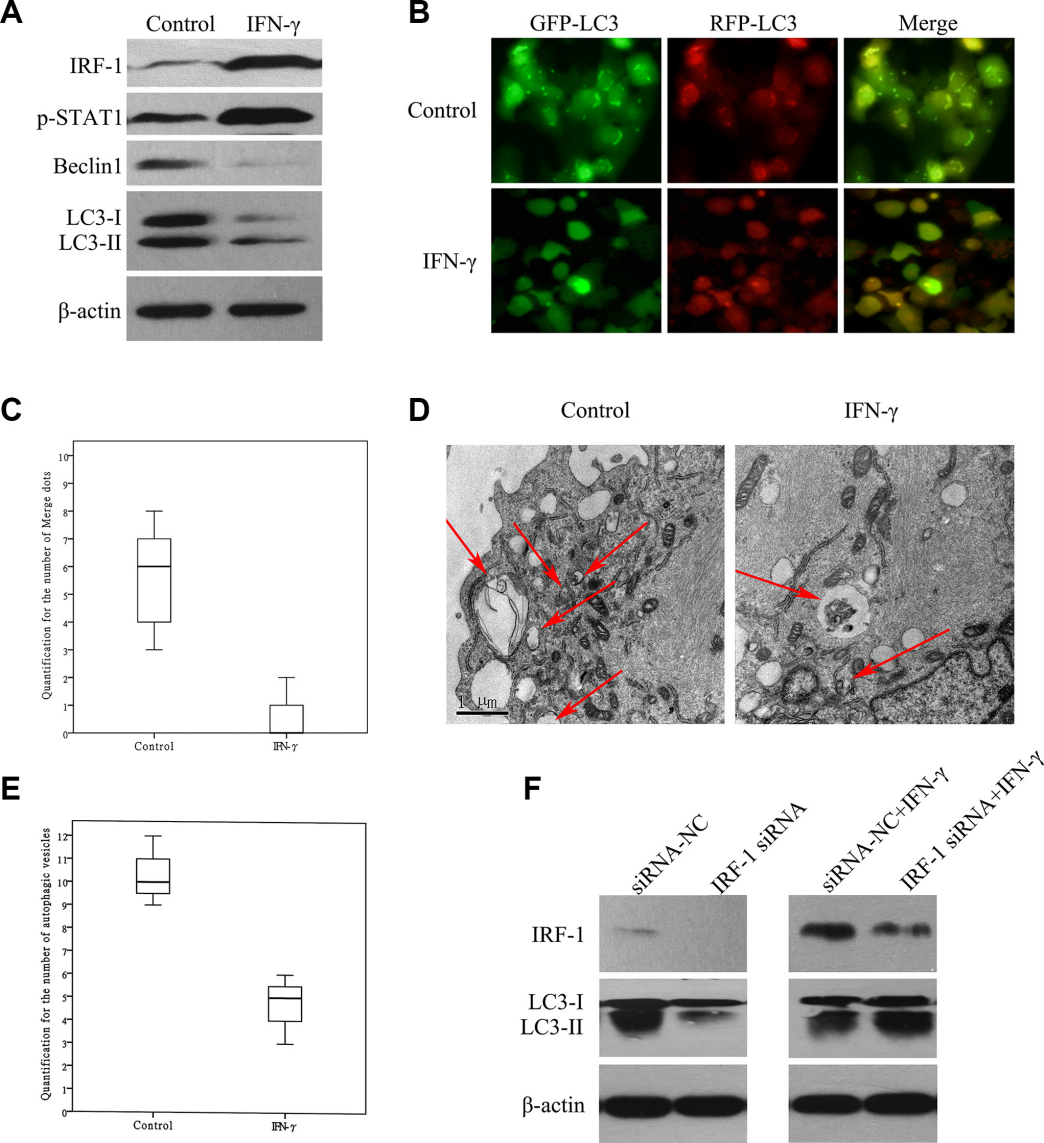
IFN-γ suppressed autophagy via IRF-1 in SK-Hep1 cells (**A**) Levels of LC3-II and Beclin1 were decreased in SK-Hep1 cells stimulated with IFN-γ. (**B**) and (**C**) In GFP-RFP-LC3 transfected SK-Hep1 cells, there was a decrease in the number of fluorescent spots after IFN-γ stimulation (*P* < 0.001). (**D**) and (**E**) A reduction of autophagosomes in the ultrastructure of SK-Hep1 cells after IFN-γ stimulation was also observed (*P* = 0.01). (**F**) After IRF-1 siRNA transfection, IFN-γ stimulated SK-Hep1 cells showed relatively lower levels of IRF-1 and higher levels of LC3-II as compared with the siRNANC+IFN-γ group. Cells in both of these two groups showed relatively higher IRF-1 levels and lower LC3-II levels than that in the siRNANC group. There was a significant reduction of LC3-II in the IRF-1 siRNA group as compared with the siRNANC group.

Results obtained using Western Blot tests demonstrated that levels of LC3-II and Beclin1 were decreased in the SK-Hep1 cells stimulated with IFN-γ as compared with the control group (Figure 4A). In Ad-GFP-RFP-LC3 transfected SK-Hep1 cells, there was a decrease in the number of fluorescent spots after IFN-γ stimulation (Figure 4B, Figure 4C, *P* < 0.001). There was also a reduction of autophagosomes in the ultrastructure of SK-Hep1 cells as observed with TEM (Figure 4D, Figure 4E, *P* = 0.01). After IRF-1 siRNA transfection, IFN-γ stimulated SK-Hep1 cells showed relatively lower levels of IRF-1 and higher levels of LC3-II as compared with that of the siRNANC+IFN-γ group (Figure 4F). Both of the IRF-1 siRNA+IFN-γ group and the siRNANC+IFN-γ group showed relatively increased IRF-1 levels and decreased LC3-II levels as compared with that of the siRNANC group.

### IFN-γ suppressed autophagy via caspase activation in SK-Hep1 cells

**Figure 5:**
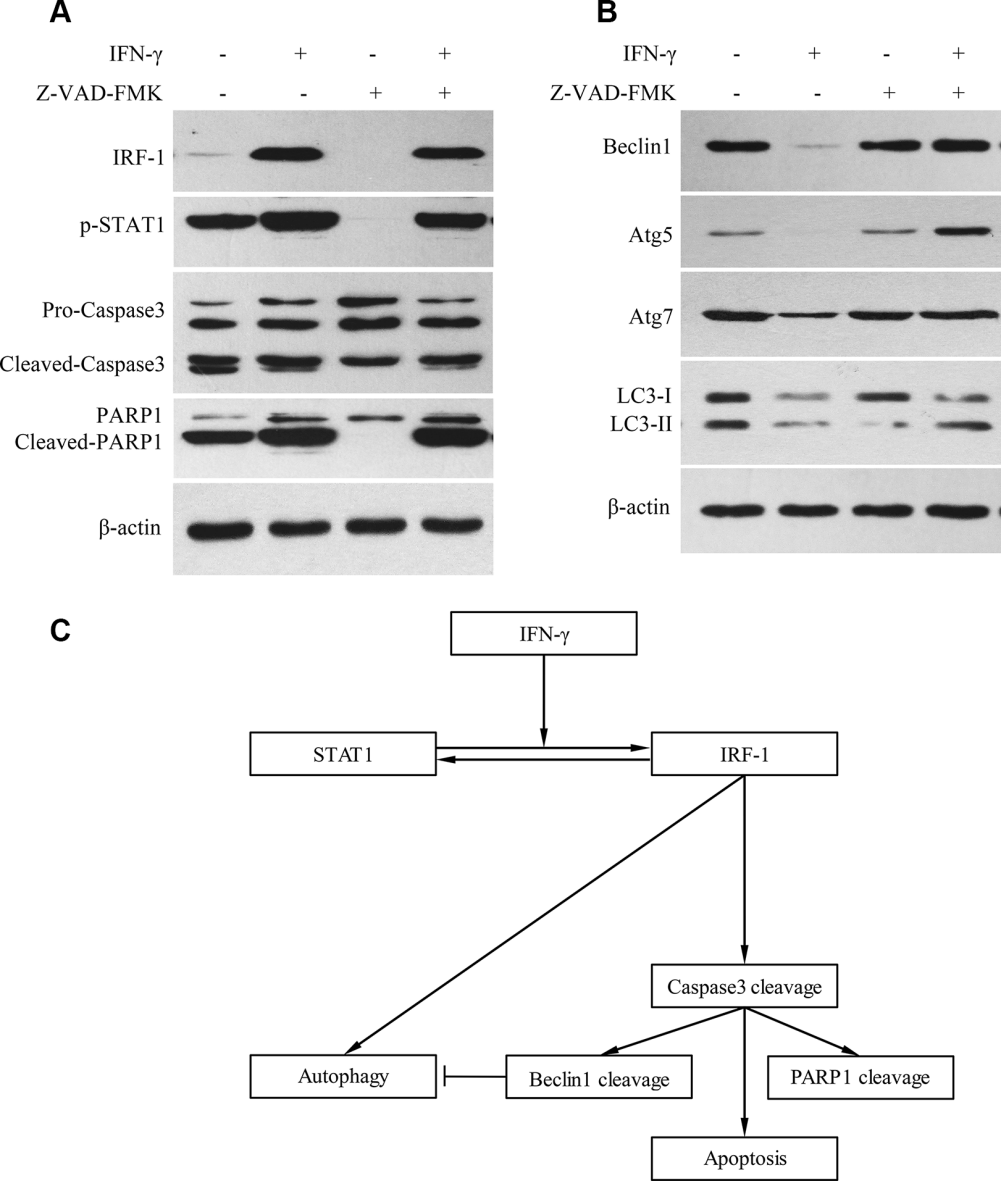
IFN-γ suppressed autophagy via caspase activation in SK-Hep1 cells (**A**) With the addition of Z-VAD-FMK, levels of cleaved Caspase-3 and PARP1 were decreased. (**B**) Levels of Beclin1, Atg5, Atg7 and LC3-II were decreased after IFN-γ stimulation. However, when IFN-γ was combined with the caspase inhibitor, levels of Beclin1, Atg5, Atg7 and LC3-II were not decreased as determined by the Western Blot test. (**C**) Summary of the roles of IRF-1 in apoptosis and autophagy.

Z-VAD-FMK is a pan caspase inhibitor. With the addition of Z-VAD-FMK, cleavage levels of Pro-Caspase-3 and PARP1 were decreased (Figure 5A). Levels of Beclin1, Atg5, Atg7 and LC3-II were also decreased after IFN-γ stimulation (Figure 5B). However, when IFN-γ was combined with Z-VAD-FMK, the levels of Beclin1, Atg5, Atg7 and LC3-II were not decreased as demonstrated using western blot tests (Figure 5B).

### Basic expression of IRF-1 was important for autophagy in SK-Hep1 cells

Results obtained with the IRF-1 siRNA transfection test revealed that IRF-1 was significantly decreased after IRF-1 siRNA transfection (Figure 4F); while in the siRNA-NC group, basic IRF-1 expression was present as demonstrated by the Western Blot test (Figure 4F). When LC-II levels were compared between the IRF-1 siRNA and siRNANC groups, a significant decrease of LC3-II was found in IRF-1 siRNA transfected cells (Figure 4F).

## DISCUSSION

A national system for organ distribution does not exist in China. As a result, a donor liver may only be allocated among a small group of candidates within several centers and occasionally there may be a candidate shortage at a center. The criterion for LT in patients with HCC has been expanded in this context, in particular given the findings that survival of LT for non-malignant diseases was reduced as based upon a high MELD score. This fact represents one of the reasons for the relatively low proportion of patients with limited HCC included in this study. A second reason is that patients with limited HCC often show a “complete necrosis” after therapies before LT and were therefore excluded from the study. HCCs at T3b and T4 stages were regarded as a contradiction for LT, however radiological grading of HCCs is difficult, when a primary spontaneous bacterial peritonitis or portal vein thrombosis was present. In this study, the HCCs of T3b and T4 were diagnosed pathologically after LT, thus portal vein and diaphragm invasions were not confirmed before transplantation. In this cohort of LT patients, HBV was the major cause of HCC. All stages of HCC were involved in our analyses. The proportion of poorly differentiated HCCs or HCCs with tumor microemboli was approximately 50% of all the cases included in our study (Table 1).

The HCC recurrence after downstaging therapy was used as a marker to predict the prognosis of LT [11–14]. HCC recurrence after hepatectomy was also reported to have an impact on the prognosis of LT [15]. However, differences in graft survival between patients with or without a history of hepatectomy can only be found in patients with advanced HCC [16]. Accordingly, the predictive value of hepatectomy before LT may be limited. In this study, the expression of biomarkers, including Ki-67, did not show any significant differences between primary and recurrent HCC (Table S1). Based upon this finding it appears that recurrent HCC would not be a confounding factor as related to comparisons of RFS.

According to our results, Ki-67 was the only biomarker that can be used for predicting RFS in LT for HCC. When comparing RFSs between different expression groups, potential confounding factors may reduce the reliability of the results. Therefore, a Cox regression model was used to substantiate the independent predictive value. Ki-67 showed a significant predictive value for HCC recurrence independent of the Milan-UCSF criteria and tumor microemboli. As HCCs of T3b and T4 stages were included in the analysis, the predictive value of Ki-67 in relatively early HCC (T1-T3a) was confirmed only within subgroup analyses (Figure 2B, *P* = 3.9 × 10^−4^). HCC at this stage was indicated for LT and Ki-67 was a likely biomarker for predicting RFS after LT. As use of the Bonferroni correction increased type II errors, factors such as BRCA1, p53 and IRF-1 may also have a predictive value with regard to HCC recurrence after LT. However, this predictive value was relatively small or not independent of the Milan-UCSF criteria, microemboli or Ki-67, indicating a limited utility for use in clinical practice.

TNM staging, a known factor for HCC recurrence was not an independent risk factor in the Cox model either. TNM staging may correlate with Milan-UCSF criteria and/or microemboli. Therefore, its independent predictive value was reduced in a model with these factors. However, the correlation between Ki-67 and TNM staging may be another explanation for it. In primary HCC, a significant correlation between Ki-67 and T stage (TNM stage) was found (Figure 3, *P* = 1.2 × 10^−5^), which showed that an increasing expression of Ki-67 was associated with HCC progression. Tumor progression may be correlated with “T stage” of primary HCC, but not with the “T stage” of recurrent lesions, due to a discrepancy in tumor progression before hepatectomy. Therefore, no correlation between Ki-67 and T stage was present in the recurrent HCC group (Figure 3, *P* = 0.501). Ki-67 should be used as a marker for HCC progression, which would serve as a valuable supplement to TNM staging.

The differences in RFS between the IRF-1 negative and positive groups did not reach statistical significance after Bonferroni correction (Figure 5A, *P* = 0.023, Bonferroni correction α′ = 1.5 × 10^−3^). According to a previous study, a significant decrease in graft survival of recurrent HCC was only found in LT for HCCs with expanded criteria [16]. Thus, the prognosis for LT may be significantly affected by behavioral factors in relatively advanced HCC, due to the low recurrence rate in early HCC. When only patients with HCC beyond the Milan criteria were studied, a significant difference in RFS was found between the negative and positive IRF-1 expression groups (Figure 5B, *P* = 6.4 × 10^−5^). In this way, IRF-1 may not be a good predictor of HCC recurrence after LT, but it may be involved in mechanisms of HCC suppression, at which time it may then provide a predictive role in relatively advanced HCC.

Ki67 is a well-known biomarker for cell proliferation, and correlates with the prognosis of malignant tumors [17–19]. IRF-1 is described as the “master promoter”, as it is involved in tumor growth regulation through a variety of mechanisms [2, 20–24]. In human Ketr-3 and 786-O renal carcinoma cells, IRF1 was found to repress Ki-67 gene transcription in a dose-dependent manner [25]. In this study, we found that a negative correlation existed between Ki-67 and IRF-1 (Figure 2D, *P* = 0.030). It has also been reported that IRF-1 and p53 exert a cooperative and independent regulation of apoptosis, depending on the type and differentiation stage of the cell [26]. Thus, apoptosis may be one of the means through which IRF-1 affects tumor growth. In this study, our *in vitro* results showed that the capacity for IFN-γ to promote apoptosis in SK-Hep1 cells was associated with increased IRF-1 and pSTAT1 levels (Figure 3). The finding that IFN-γ promotes apoptosis via IRF-1 has been supported by other studies [27, 28], and we believe that IRF-1 is also involved in IFN-γ induced apoptosis in SK-Hep1 cells.

Interestingly, there is a report indicating that in certain HCC cells IFN-γ was found to induce autophagy instead of apoptosis through the IRF-1 signaling pathway [10]. However, this role of IRF-1 on autophagy has not been consistently demonstrated in HCC cells. In this study, we examined the potential for autophagy in SK-Hep1 cells as assessed under several different conditions (Figure 4). We found that levels of LC3-II, Beclin1, Atg5 and Atg7 were all decreased in SK-Hep1 cells stimulated with IFN-γ (Figure 4A, Figure 5B). There were also a significant reduction in the number of fluorescence spots of LC3 and autophagosomes (Figure 4B–4E). These results indicate that a reduction in autophagy is associated with apoptosis activation. As caspase activation is crucial for apoptosis, we further analyzed the role of caspase activation on autophagy, with use of the caspase inhibitor, Z-VAD-FMK. Levels of Beclin1, Atg5, Atg7 and LC3-II were not decreased in SK-Hep1 cells, when IFN-γ and caspase inhibitor were added together (Figure 5B). Thus, caspase likely plays a role in suppressing autophagy. After IRF-1 siRNA transfection and IFN-γ stimulation, SK-Hep1 cells showed relatively higher levels of LC3-II, in comparison with that of SK-Hep1 cells transfected with siRNA-NC and stimulated with IFN-γ (Figure 4F). Therefore, IRF-1 expression and caspase activation both appear to be involved in autophagy suppression in response to IFN-γ stimulation, which may represent the cross-talk that exists between apoptosis and autophagy (Figure 5C). Beclin1 was reported to be cleaved by Caspase-3, which then inactivates autophagy and promotes apoptosis [29]. Based on our current results, other proteins, such as Atg5 and Atg7, which are involved with autophagy, may also be reduced by caspase.

In addition to IRF-1's contribution to apoptosis, the effect of IRF-1 expression on autophagy was also studied using IRF-1 siRNA. In our experiment with siRNA transfection, there was a significant decrease in the level of LC3-II after IRF-1 siRNA transfection (Figure 4F). We conclude that a basic expression of IRF-1 seems to be important for autophagy (Figure 5C). IFN-γ induced autophagy was also found in another HCC cell line, Huh-7 [10], a cell line that was not sensitive to IFN-γ induced apoptosis. Therefore, IRF-1 appears to promote autophagy in the absence of caspase activation.

In conclusion, the capacity for IRF-1 involvement in apoptosis and autophagy results from different mechanisms. As an upstream promoter, IRF-1 may be used as a potential target for HCC treatment, based on its capacity to affect apoptosis and autophagy. In addition, Ki-67 shows great promise for the prediction of HCC recurrence in LT and can be used to aid in the selection of LT candidates.

## MATERIALS AND METHODS

### Clinical study

#### Patients

An ambispective cohort study of LT recipients was conducted in Tianjin First Central Hospital. Patients receiving LT between Jan 1st 2012 and Dec 31st 2014 were involved in this study. Final follow-up was conducted on Dec 31st 2015. The inclusion criteria of patients for this study included adult patients that: (1) were greater than 18 years of age with a distinctive diagnosis of HCC before transplantation, (2) underwent primary LT, (3) received full liver grafts and (4) possessed histologically intact HCC tissue (for immunohistochemical staining).

### Management

The immunosuppressive regimen consisted of tacrolimus, mycophenolate mofetil, steroids and basiliximab. All the patients had liver cirrhosis and granulocytopenia, indicating that chemotherapy was not a routine treatment after LT. Immunosuppression reduction and early treatments for recurrent HCC were employed. Patients underwent CT scan every 1–3 months and monthly Ultrasounds after LT.

The study was approved by the Tianjin First Central Hospital Research Ethics Committee. Tumor specimens used in our analysis were from the tissue bank of Tianjin First Central Hospital. Informed consent was obtained from each patient before specimen removal and storage in the tissue bank. Base line data on the patients and their tumors were recorded.

### Definitions and grouping

Among patients with intact HCC lesions before LT, the complete or portions of the original tumor lesion was classified as “primary HCC”, including patients with residual lesions after radiofrequency (RF) or transcatheter hepatic arterial chemoembolization (TACE). Patients with recurrent tumor lesions after hepatectomy were classified as “recurrent HCC”. The use of RF and TACE, to reduce tumor size, were usually performed at 1 month before LT, thus there were no recurrent HCCs after RF or TACE.

Tumor recurrence after LT referred to the first diagnosis made by Ultrasound, MRI, CT or PET-CT. Patient death was declared by a doctor during hospitalization in our center or confirmed by a following-up phone call (to a patient's relative) when a patient missed their date of examination. Time to recurrence (TTR) was defined by the time interval between the date of LT and the first observation of lesion recurrence, and was confirmed upon final diagnosis of HCC recurrence as determined by a subsequent examination. The recurrence-free survival time of patients without recurrence was defined by the date of their last chest-abdomen CT, which was used as the censor data of Kaplan-Meier survival.

Tumor staging, differentiation and microemboli were determined by the pathological presentations of the HCC within resected liver after LT. (Tumor staging was determined only by the recurrent lesions in patients with hepatectomy history prior to LT). Tumor staging was defined according to the sixth edition of TNM classification and the Milan and UCSF criteria. Tumor differentiation was graded according to the Edmondson grading system (2010).

It was difficult to determine expression levels when the immunohistochemical staining result was “+”. Therefore, two grouping methods were employed in the analysis: 1) Negative/positive grouping - a result of “−” was assigned to the negative group while results of “+”, “++” or “+++” were assigned to the positive group and 2) Low/high grouping - results of “−” or “+” were assigned to the low group while results of “++” or “+++” were assigned to the high group.

### Main outcome measures

The RFS was the main outcome measure.

### Laboratory method

#### Cells, antibodies and reagents

The SK-Hep1 cell line used in this study was from the cell bank of the Chinese Academy of Sciences. Cells were cultured in DMEM containing 10% heat-inactivated fetal bovine serum (FBS).

The rabbit anti-human monoclonal antibodies (CST) of CK, CK19, GPC3, AFP, VEGF, EGFR, ERCC1, RRM1, TYMS, BRCA1, p53, Vimentin, pSTAT1, Ki-67, Caspase-3, Beclin1, Atg5, Atg7, LC3A/B and IRF-1 were used for immunohistochemical staining or western blot tests. β-actin (CST) was used as an internal control in the western blot test. The DAPI and PI Staining Solution were from Sigma Co. Annexin V-FITC/PI kit (BD) was used for cell staining before flow cytometry assay.

IFN-γ (Peprotec) was used to activate the expression of IRF-1. IRF-1 siRNA (Guangzhou Ribobio Co.) was used to reduce the expression of IRF-1 and siRNA-NC (Guangzhou Ribobio Co.) was used as a negative control condition. Z-VAD-FMK (Selleck) was used to inhibit activated caspase. The anti-sense sequence of IRF-1 siRNA was 5′- dTdT CUUUCUUUCAGCUUCAGGU-3′. The siRNA was transfected with lipo2000 (Invitrogen) according to the manufacturer's instructions. The ad-RFP-GFP-LC3 adenovirus (Shanghai Hanbio Co.) was used to introduce RFP-GFP-LC3 into SK-Hep1 cells.

### Immunohistochemical staining

Immunohistochemical staining was performed using a DAB detection kit (Beijing ZSGB-Bio Co.). Briefly, after dewaxing, hydration and microwave antigen retrieval, slides were blocked with rabbit serum. Slides were then incubated overnight with the primary antibody (1:500) (CK, CK19, GPC3, AFP, VEGF, EGFR, ERCC1, RRM1, TYMS, BRCA1, p53, Vimentin, Ki-67 or IRF-1). Biotin-conjugated secondary antibodies (1:500, goat anti-rabbit, Beijing ZSGB-Bio Co.) and strept avidin-biotin complex (SABC) were used for immunohistochemical staining.

An index for each view of immunochemical staining was calculated by multiplying the score for intensity of color stained in the cytoplasm/nucleus and the score for proportion of tumor cell cytoplasm/nucleus stained. The score of intensity was defined as none = 0, light yellow = 1, brownish yellow = 2, and brown = 3; while the definition of scores for proportions were 0–25 % = 1, 26–50 % = 2, 51–75 % = 3 and 76–100 % = 4. An average index was then calculated as resulting from the index of 5 randomly selected views. Finally 4 grades (Table S1) were determined by the average index: 0 = “−”, 1–4 = “+”, 5–8 = “++”, 9–12 = “+++”.

### Autophagosomes quantification

SK-Hep1 cells were cultured with RFP-GFP-LC3 adenovirus (Shanghai Hanbio Co.) After Ad-RFP-GFP-LC3 was introduced into SK-Hep1 cells, these cells were cultured normally or stimulated with IFN-γ (100 ng/ml, Peprotec) for 24 h. Then double labeled SK-Hep1 cells were observed under a fluorescence microscope (LX81, OLYMPUS). This experiment was conducted for 3 times in each group. Total 6 merge images were used for quantification. From up to down, 10 well-recognized cells were selected in each merge image, and the number of fluorescent spots in each cell was counted by observing. The numbers of fluorescent dots in cells were compared between groups.

Ultrastructures of SK-Hep1 cells were studies under a transmission electron microscope (TEM 100CX, JEOL). 3 SK-Hep1 cells were randomly selected in each time of TEM observation and the number of autophagosomes in each cell was determined by observing. Then a mean number of autophagosomes in one cell was calculated in each time of TEM observation. This experiment was conducted for 3 times in both groups. The mean numbers of autophagosomes were compared between groups.

### Cellular viability analysis

Cell viability was detected with use of a MTT kit (Sigma). Cells in the negative control group and the control group were cultured normally. Cells in the IFN-γ group were stimulated with IFN-γ (100 ng/ml, Peprotec). In each group, cells were plated and cultured with or without IFN-γ for 0, 12, 24, 36 or 48 h. The MTT solution (5 mg/ml, 10μl) was added and removed 4 h later. Spectrophotometric values were measured at a wavelength of 570 nm after the solvent was added. The survival ratios of cells in the control group and in the IFN-γ group were calculated by the following methods. survival ratio(control) = Absorbance(control)/Absorbance(negative control); survival ratio(IFN-γ) = Absorbance(IFN-γ)/Absorbance(negative control).

### DAPI/PI staining

SK-Hep1 cells(2 × 10^4^ cells) were planted and cultured with or without IFN-γ (100 ng/ml, Peprotec) for 24 h. Then the PI (50 μM, Sigma) solution was added for 20 min and DAPI (5 μg/ml, Sigma) solution was added for 10 min. Cells were observed under a fluorescence microscope (IX81, OLYMPUS).

### Annexin V-FITC/PI and flow cytometry assay

After SK-Hep1 cells were cultured with or without IFN-γ (100 ng/ml, Peprotec) for 24 h. Cells(1 × 10^6^ cells in 100 μl) were stained with Annexin V-FITC (Annexin V-FITC/PI kit, BD) for 10 min and with PI Staining Solution (Annexin V-FITC/PI kit, BD) for 15 min in turn. The proportions of stained cells were evaluated by a FCM flow cytometer (BD Accuri C6).

### Cell culture and treatment before western blot analysis

Cells were plated and treated with reagents or siRNA (IFN-γ, Z-VAD-FMK or siRNA) according to their group: 1) Cells (SK-Hep1 cells or ad-RFP-GFP-LC3 transfected SK-Hep1 cells) were cultured for 24 h in the control group and then cultured with IFN-γ (100 ng/ml, Peprotec) for 24 h for the IFN-γ group, 2) siRNA-NC and IRF-1 siRNA were transfected into SK-Hep1 cells in the siRNANC and siRNA groups, respectively, and cultured for 72 h. In the siRNA-NC+IFN-γ or siRNA+IFN-γ group, siRNA-NC or siRNA transfected SK-Hep1 cells were recovered after 48 h and then cultured with cell media containing IFN-γ (100 ng/ml, Peprotec) for 24 h and 3) The same treatments as described for group 1 were used to establish the control and IFN-γ groups. In the Z-VAD-FMK group, SK-Hep1 cells were cultured with Z-VAD-FMK (50 nM, Selleck) for 24 h. In the Z-VAD-FMK+IFN-γ group, SK-Hep1 cells were cultured with a combination of IFN-γ (100 ng/ml, Peprotec) and Z-VAD-FMK (50 nM).

### Western blot analysis

The Western Blot test was performed as described in previous reports [30]. Proteins were extracted from the HCC cell line SK-Hep1 and separated by SDS-PAGE. After proteins were transferred to polyvinylidene fluoride membranes (Millipore), each protein was detected with its antibody, including IRF-1 (1:1500, CST) and LC3A/B antibodies (1:1500, CST). β-actin was used as an internal control.

### RT-PCR

A kit (TaKaRa) was used for RT-PCR. Total RNAs were isolated with Trizol reagent. RNA (2 μg) was used in the reverse transcription and followed by PCR procedures. The PCR products were examined with agarose gel electrophoresis. GAPDH was used as the internal control in RT-PCR. The sequence of upstream primer for Beclin1: 5′-AGGAGAGACCCAGGAGGAAG-3′; the sequence of downstream primer for Beclin1: 5′-GGCACT TTCTGTGGACATCA-3′; the sequence of upstream primer for GAPDH: 5′-CAGCCAGGAGAAATCAAACAG-3′; the sequence of downstream primer for GAPDH: 5′-GACT GAGTACCTGAACCGGC-3′.

### Statistical analysis

All data were analyzed using SPSS 18.0 (SPSS, Inc., Chicago, IL, USA). Numerical data were compared using the Pearson Chi-Square, continuity correction of Chi-Square or likelihood ratio test. The one-sample Kolmogorov-Smirnov test was employed to determine the distribution of the data. Data with normal distributions were presented as “means ± SDs” and compared by either Student *t*-tests or one-way analysis of variance (ANOVA). Data failing to show a normal distribution were presented as “medians and ranges” and were compared using the Mann-Whitney *U*-test. The Spearman correlation was used to evaluate the relationship between data without a normal distribution. RFSs were estimated by the Kaplan-Meier method and compared with use of the log rank test. The Cox regression analysis was used to confirm independent risk factors for HCC recurrence. A *p* < 0.05 was required for results to be considered statistically significant. A total of 33 comparisons involving survivals were conducted between different expression groups of biomarkers, including a subgroup comparison. The Bonferroni correction was used to adjust for an accumulated type I error with multiple comparisons (α′ = 1.5 × 10^−3^). Analyses of distributions or correlations as well as the subgroup analyses of a positive overall result, were not corrected for α value (α = 0.05).

Laboratory tests were repeated three times. The relative levels of cell viability and numbers of autophagosomes were compared by ANOVA. Numbers of fluorescent spots were compared by Mann-Whitney *U*-test. A *p* < 0.05 was required for results to be considered statistically significant.

## SUPPLEMENTARY MATERIALS TABLES


