# Supplementary material for: Bi-directional roles of IRF-1 on autophagy diminish its prognostic value as compared with Ki67 in liver transplantation for hepatocellular carcinoma
Source: Oncotarget. 2016 May 13;7(25):37979–92. doi: 10.18632/oncotarget.9365 (PMC5122365; doi:10.18632/oncotarget.9365)
Supplement: Supplementary file 1 [file oncotarget-07-37979-s001.pdf]

Bi-directional roles of IRF-1 on autophagy diminish its prognostic value as compared with Ki67 in liver transplantation for hepatocellular carcinoma

Supplementary Materials

Supplementary Table S1: Immunochemical results of biomarker in primary and recurrent HCCs

	Primary HCC	Recurrent HCC	Parameter	P
CK[a]			LR = 0.979	0.613
−	9	3		
+	25	4		
++	68	18		
CK19[a]			LR = 0.226	0.893
−	80	19		
+	13	3		
++	9	3		
GPC3[a]			LR = 4.232	0.238
−	10	3		
+	31	5		
++	61	16		
+++	0	1		
AFP[a]			$\chi^2 = 0.269$	0.874
−	42	11		
+	21	4		
++	39	10		
VEGF[a]			LR=1.358	0.716
−	41	11		
+	35	7		
++	24	7		
+++	2	0		
EGFR[a]			LR = 2.827	0.419
−	37	12		
+	21	2		
++	35	9		
+++	9	2		
ERCC1[b]			LR = 1.377	0.711
−	70	18		
+	13	3		
++	18	3		
+++	1	1		
RRM1[a]			LR = 2.899	0.408
−	17	6		
+	30	8		

++	50	11		
+++	5	0		
TYMS [a]			LR = 2.351	0.309
−	73	14		
+	18	6		
++	11	5		
BRCA1 [b]			LR = 2.344	0.504
−	73	20		
+	12	2		
++	16	2		
+++	1	1		
p53 [b]			LR = 7.218	0.065
−	68	11		
+	16	3		
++	14	9		
+++	4	2		
VIM [a]			LR=5.932	0.115
−	54	13		
+	15	1		
++	33	10		
+++	0	1		
Ki-67 [b]			LR = 1.128	0.770
−	52	13		
+	30	8		
++	15	2		
+++	5	2		
IRF-1			$\chi^2 = 5.526$	0.063
negative	36	13		
Plasma positive	51	6		
Nucleus positive	15	6		

a: Determined by expression in cytoplasm or membrane;
b: Determined by expression in nucleus;
LR: Likelihood Ratio.

Supplementary Table S2: Bonferroni correction for comparisons of RFSs

Rank	Grouping method	*p* value	Log Rank χ^2	Significant*
1	Milan and UCSF criteria[a]	3.8×10^{-14}	61.803	Yes
2	With/without tumor microemboli	1.7×10^{-11}	45.291	Yes
3	Ki-67 negative/positive	4.6×10^{-5}	16.589	Yes
4	IRF-1 cytoplasm negative/positive (Beyond Milan criteria Subgroup)	6.4×10^{-5}	15.980	Yes
5	Ki-67 low/high expression	1.6×10^{-4}	14.268	Yes
6	p53 negative/positive	0.009	6.740	No
7	IRF-1 cytoplasm negative/positive	0.023	5.167	No
8	Low/high differentiation	0.024	5.113	No
9	BRCA1 low/high expression	0.038	4.322	No
10	CK low/high expression	0.102	2.680	No
11	TYMS low/high expression	0.105	2.622	No
12	ERCC1 low/high expression	0.162	1.955	No
13	VEGF negative/positive	0.225	1.472	No
14	AFP low/high expression	0.231	1.436	No
15	GPC3 low/high expression	0.233	1.423	No
16	IRF-1 nuclear negative/positive	0.244	1.358	No
17	BRCA1 negative/positive	0.410	0.678	No
18	CK negative/positive	0.414	0.668	No
19	VIM low/high expression	0.440	0.596	No
20	EGFR negative/positive	0.444	0.587	No
21	ERCC1 negative/positive	0.503	0.448	No
22	AFP negative/positive	0.518	0.417	No
23	GPC3 negative/positive	0.547	0.364	No
24	EGFR low/high expression	0.612	0.257	No
25	VIM negative/positive	0.711	0.137	No
26	RRM1 low/high expression	0.767	0.088	No
27	p53 low/high expression	0.778	0.079	No
28	CK19 low/high expression	0.852	0.035	No
29	CK19 negative/positive	0.864	0.029	No
30	TYMS negative/positive	0.891	0.019	No
31	Primary/recurrent HCC	0.896	0.017	No
32	RRM1 negative/positive	0.927	0.009	No
33	VEGF low/high expression	0.951	0.004	No

a: Cases were stratified into 3 levels: within Milan criteria (level 1), between Milan and UCSF criteria (level 2) and beyond UCSF criteria (level 3);
*: Bonferroni correction, $\alpha' = 1.5 \times 10^{-3}$.
